# Molecular, Histologic, and Radiologic Findings of High-Grade Invasive Adenocarcinoma Arising in Oncocytic Subtype of Intraductal Papillary Mucinous Neoplasm: A Case Report and Review of Literature

**DOI:** 10.1089/pancan.2016.0017

**Published:** 2017-02-01

**Authors:** Jared Shows, Christan Bartsch, Heather Carmichael, Irfan Qureshi, Barish Edil, Hubert Fenton

**Affiliations:** ^1^ Department of Pathology, University of Colorado Denver School of Medicine, Aurora, Colorado.; ^2^ Department of Pathology, Children's Hospital of Los Angeles, Los Angeles, California.; ^3^ Department of Surgery, UC Health Anschutz Medical Campus, Aurora, Colorado.

**Keywords:** intraductal, intraductal papillary mucinous neoplasm, KRAS, oncocytic, oncocytic papillary neoplasm

## Abstract

**Background:** We present a case of adenocarcinoma arising in the oncocytic subtype of intraductal papillary mucinous neoplasm (O-IPMN), with emphasis on the molecular findings in the adenocarcinoma component. Tissue microdissection and next-generation sequencing were performed using a 26 gene panel (*AKT1, ALK, APC, BRAF, CDH1, CTNNB1, EGFR, ERBB2, FBXW7, FGFR2, FOXL2, GNAQ, GNAS, KIT, KRAS, MAP2K1, MET, MSH6, NRAS, PDGFRA, PIK3CA, PTEN, SMAD4, SRC, STK11, TP53*) of cancer-related genes.

**Case Presentation:** A 69-year-old Caucasian female presented with chest pain and was found to have findings consistent with acute pancreatitis. During her work-up, computed tomography scan revealed a large cystic and solid mass in the tail of the pancreas. She recovered from her acute pancreatitis and was discharged home. She later returned for resection of her mass.

**Results:** Evaluation of three microdissected regions of tumor demonstrated no identifiable nonsynonymous alterations in any of the three regions, within the targeted genes.

**Conclusion:** This case demonstrates that the O-IPMN is a molecularly distinct subtype, and we conclude that adenocarcinoma arising in these neoplasms shows molecularly distinct tumorigenesis from traditional pancreatic ductal adenocarcinoma. These differences may help explain the improved survival with invasive adenocarcinoma arising from these lesions compared with traditional ductal adenocarcinoma.

## Introduction and Background

Intraductal papillary mucinous neoplasms (IPMNs) are a heterogeneous group of tumors. The epithelial subtypes (gastric, intestinal, pancreatobiliary, and oncocytic) have been shown to be independent predictors of biological behaviors, recurrence, and long-term survival rate. The rarest subtype, oncocytic, was first described by Adsay et al.^1^ These lesions tended to be larger than the typical IPMN, measuring up to 15 cm in size, with no predilection for location in the pancreas. Infrequently, these tumors develop an associated low-grade invasive carcinoma that retains the oncocytic features. With the introduction of routine molecular genetic analysis, including next-generation sequencing (NGS), recent studies have shown that these lesions are, in fact, genetically distinct from the typical IPMNs, which likely contribute to prognostic differences. We present a unique case of a 69-year-old female with a high-grade signet ring cell carcinoma arising within an oncocytic IPMN (O-IPMN), with associated radiological studies, molecular testing, and a review of literature.

## Presentation of Case

The patient was a 69-year-old Caucasian, otherwise healthy female who initially presented to an outside hospital with chest pain and was found to have an elevated serum lipase consistent with acute pancreatitis. Notably, she had a history of pancreatitis 16 years prior, thought to be secondary to gallstones, and underwent cholecystectomy at that time. During her work-up for pancreatitis, computed tomography (CT) scan revealed a large, multilobular mass in the tail of the pancreas; the mass had mixed cystic and solid components, which is concerning for a mucinous cystic neoplasm. Follow-up magnetic resonance imaging (MRI) corroborated CT findings. She recovered from her acute pancreatitis during an inpatient stay and was ultimately discharged home. After discharge, the patient remained largely asymptomatic.

She was referred to the University of Colorado Hospital Pancreas Multi-Disciplinary Clinic for evaluation and surgical management. A complete blood count and complete metabolic panel were within normal limits. Carcinoembryonic antigen and carbohydrate antigen 19-9 were also within normal limits. Imaging studies were reviewed and management discussed with more than 20 physicians from a broad range of specialties. Ultimately, given the radiological appearance of the lesion and concern for high-grade dysplasia versus invasive carcinoma, the recommendation was made to proceed with resection. The patient underwent open distal pancreatectomy and splenectomy.

### Surgical findings

The patient was taken to the operating room for distal pancreatectomy and splenectomy. Upon exploration, there was no evidence of metastatic disease or lymphadenopathy. Therefore, it was decided to proceed with planned distal pancreatectomy and splenectomy. The entire tail of the pancreas was easily exposed, revealing a softball-sized cystic neoplasm along the inferior edge of the pancreatic hilum. It was a well-circumscribed cyst without any inflammation. The rest of the pancreas was soft with normal architecture. It was a very classic appearance of a cystic neoplasm. The spleen was then mobilized into the operative field easily, and the retroperitoneal attachments extending to the ligament of Treitz were easily taken down. The splenic artery and splenic vein were then encircled and ligated. The pancreatic neck was then thinned out and stapled across with an Endo-GIA stapler. The specimen was sent to pathology with negative margins. The procedure was well tolerated by the patient and without complications. Estimated blood loss was 100 cc.

### Postoperative care

Her postoperative course was unremarkable, and she was discharged home 6 days later. Intraoperative pancreatic margin was negative for malignancy. Final pathology showed an intraductal oncocytic papillary neoplasm (IOPN) measuring 11 × 9 × 7 cm, with a component of invasive adenocarcinoma. Microscopic margins, as well as 17 sampled lymph nodes, were negative for malignancy. The invasive component of the tumor was difficult to assess owing to extensive necrosis, but was 2.2 cm in greatest diameter for the viable portion of the tumor.

### Imaging findings

**FIG. 1.**
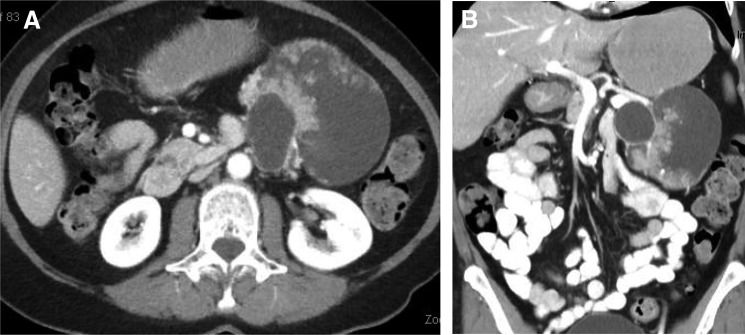
Radiographic appearance of cystic neoplasm in the tail of the pancreas. **(A)** Transverse and **(B)** coronal image of cystic neoplasm arising from the tail of the pancreas with solid and cystic components.

Initial CT scan showed a multilocular, mixed solid, and cystic mass, which measured 9.0 cm in the greatest dimension (Fig. 1). Follow-up MRI was consistent with a large, lobulated, complex cystic, and solid lesion arising from the tail of the pancreas, which measured 7.0 × 9.7 × 10.4 cm. Throughout the lesion, there were multiple enhancing mural nodules. We observed communication with the pancreatic tail measuring 3 mm in diameter, but otherwise noted no pancreatic ductal dilation. There was no appreciable lymphadenopathy or other metastasis.

### Pathological findings

**FIG. 2.**
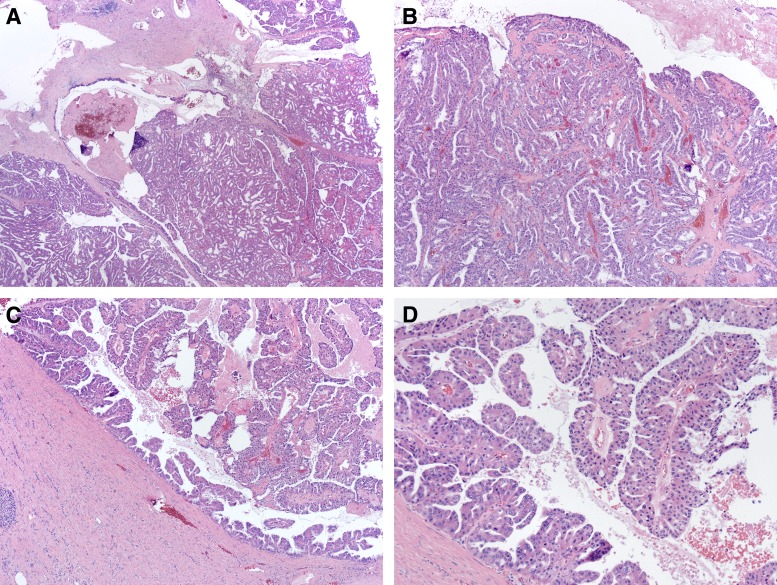
Low power of the neoplasm shows an arborizing papillae with fibrovascular cores filling a cystic space **(A, B)**. Higher power shows oncocytic tumor cells defined by granular, eosinophilic cytoplasm, and cleared nuclei with apparent nucleoli **(C, D)**.

**FIG. 3.**
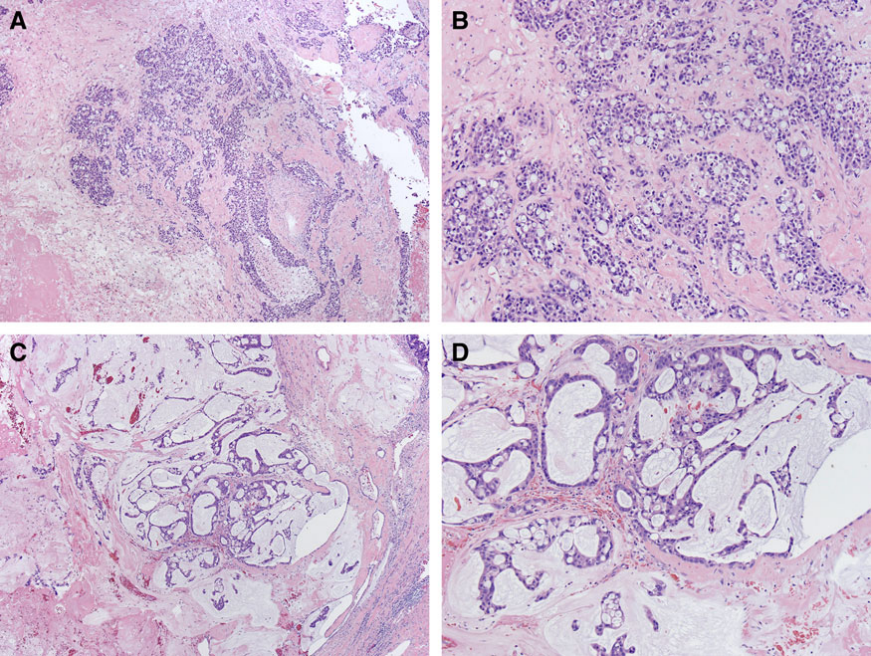
Other sections of the tumor show a more invasive pattern of growth **(A)**. Higher power view of the tumor cells show areas that take on signet ring histology **(B)** as well as areas taking on mucinous adenocarcinoma histology **(C, D)**.

The distal pancreatectomy specimen contained a well-encapsulated 11-cm multilocular, cystic lesion, which caused dilation of the main pancreatic duct. The cyst contained watery mucin contents and numerous solid-to-papillary excrescences. Microscopically, the cysts were composed of edematous, paucicellular stroma lined by simple columnar cells with abundant granular eosinophilic cytoplasm and prominent nucleoli with scattered goblet cells. These cells formed complex arborizing papillae, cribriform patterns, and varying degrees of solid growth. Multiple foci of low-grade and high-grade invasive carcinoma were present. The low-grade areas were characterized by small nests of tumor cells within abundant extracellular mucin. The high-grade areas were composed of signet ring cells with invasion into the stroma. Cytologically, the low-grade tumor displayed the same densely eosinophilic cytoplasm of the cystic lining (Figs. 2 and 3). No extra pancreatic extension was identified, and no metastatic carcinoma was identified in 17 lymph nodes.

### Molecular testing

Typically, well-documented events are responsible for the biological behavior of pancreatic ductal carcinomas. Most cases show inactivation of tumor suppressor genes *CDKN2A*, *TP53*, and *MADH4,*^2^ and activation of the protooncogene *KRAS*. To evaluate the invasive component of this tumor for these recognized molecular abnormalities, NGS was performed.

Ten-micron cut sections were deparaffinized, hematoxylin counterstained, and microdissected by scalpel point under a microdissecting microscope. The microdissected material was washed with 70% ethanol, air dried, and resuspended in lysis buffer, and DNA was extracted (Qiagen QIAamp DSP DNA FFPE Tissue Kit) using manual extraction with elution into 30 μL of elution buffer. Library preparation was performed through the Illumina TruSight Tumor 26 kit per the manufacturer's instructions (with minor modifications). This kit amplifies selected regions of 26 cancer-related genes: *AKT1, ALK, APC, BRAF, CDH1, CTNNB1, EGFR, ERBB2, FBXW7, FGFR2, FOXL2, GNAQ, GNAS, KIT, KRAS, MAP2K1, MET, MSH6, NRAS, PDGFRA, PIK3CA, PTEN, SMAD4, SRC, STK11,* and *TP53.* Libraries were sequenced on the Illumina MiSeq platform for a targeted depth of no less than 500 × for any individual amplicon. A custom-built bioinformatics pipeline utilizing GSNAP for sequence alignment and FreeBayes for variant calling was employed for data analysis. All genomic regions were verified to be covered by at least 500 sequencing reads, and identified variants were manually inspected using Integrative Genomics Viewer (Broad Institute).

## Results

Evaluation of three separately microdissected regions of the tumor demonstrated no identifiable nonsynonymous alterations in any of the three regions, within the targeted areas of our cancer-related gene panel.

## Discussion and Literature Review

The formerly named IOPN is an exceedingly rare lesion that has been recently reclassified as an O-IPMN.^3^ Recent molecular studies suggest that, when strict morphological criteria are applied, the oncocytic subtypes are genetically distinct lesions. These lesions lack the most common molecular abnormalities found in IPMNs. Specifically lacking are *KRAS* mutations that have been detected in the majority of IPMNs,^4–6^ activating *GNAS* mutations at codon 201 that have been identified in approximately half of IPMNs,^7^ and inactivating mutations in the *RNF43* gene, a tumor suppressor in the Wnt signaling pathway. Instead, molecular studies have shown somatic mutations in *ARHGAP26, ASXL1, EPHA8*, and *ERBB4*. The differences suggest that these oncocytic subtypes have a distinct pathway of tumorigenesis that likely contributes to the relatively indolent nature of the lesion. In addition, genetic testing of the associated adenocarcinoma has failed to demonstrate *CDKN2A*, *TP53*, *MADH4,*^7^ or activation of the protooncogene *KRAS* found in the majority of pancreatic ductal adenocarcinomas.

Our case of high-grade adenocarcinoma arising in association with an O-IPMN is morphologically unique in that all of the documented invasive tumors associated with O-IPMNs exhibit low-grade morphology with or without extracellular mucin. Our tumor is composed of low-grade elements but also contains areas of high-grade, signet ring morphology. Although this high-grade morphology might suggest some homology with invasive ductal adenocarcinoma, NGS failed to show similar molecular changes. This provides additional evidence that O-IPMNs have a distinct genetic tumorigenesis. This may explain why, empirically, these lesions have an improved 5-year survival compared with gastric, pancreatobiliary, or intestinal IPMNs^1^ with or without an invasive component.

## Abbreviations Used

CT: computed tomography
IOPN: intraductal oncocytic papillary neoplasm
IPMNs: intraductal papillary mucinous neoplasms
MRI: magnetic resonance imaging
NGS: next-generation sequencing
O-IPMN: oncocytic subtype of IPMN
